# Paying the Toll in Nuclear Reprogramming

**DOI:** 10.3389/fcell.2017.00070

**Published:** 2017-08-17

**Authors:** Chun Liu, Farhan Himmati, Nazish Sayed

**Affiliations:** ^1^Stanford Cardiovascular Institute, Stanford University School of Medicine Stanford, CA, United States; ^2^Department of Medicine, Stanford University School of Medicine Stanford, CA, United States

**Keywords:** nuclear reprogramming, human induced pluripotent stem cells, transdifferentiation, innate immunity, toll-like receptors

## Abstract

The ability to reverse lineage-committed cells toward pluripotent stem cells or to another cell type is one of the ultimate goals in regenerative medicine. We recently discovered that activation of innate immunity, through Toll-like receptor 3, is required during this conversion of cell fate by causing global changes in the expression and activity of epigenetic modifiers. Here we discuss, in a comprehensive manner, the recent studies on the role of innate immunity in nuclear reprogramming and transdifferentiation, the underlying mechanisms, and its role in regenerative medicine.

## Introduction

For decades, researchers have tried to turn back the cellular clock. Until the 1950s, it was generally believed that cells lost part of their genome as they differentiated into terminal cells in order to maintain their specialization, such as B-lymphocytes. However, Dr. John Gurdon's pioneering work using the *Xenopus* model system, where he showed that live adults could still be created using the nuclei of differentiated cells (Gurdon, 1962), laid the important groundwork for modern stem cell biology. Since his initial discovery, many researchers have attempted to perform nuclear transfers in mammals, including the famous “Dolly, the sheep”(Campbell et al., 1996; Wilmut et al., 1997). Similarly, the discovery that MyoD, a mammalian transcription factor that can convert fibroblasts into myocytes led to the understanding that master regulators can determine lineage specification (Davis et al., 1987). These developments were landmarks in biological research, with the potential to uncover many unknown areas in genetics and medicine.

## Embryonic stem cells

During this time when many mammals were being cloned by somatic cell nuclear transfer, Thomson and co-workers were able to successfully derive the first embryonic stem cell line (ESCs) from a human embryo (Thomson et al., 1998). This allowed researchers to understand for the first time the genetic framework that kept a cell in an embryonic state or the changes the cell underwent during the differentiation process. Although this had far reaching implications for medical research, it also raised the prospects that ESCs could be used for human cloning, at the same time generated several ethical concerns. Additionally, the low efficiency of nuclear transfer in humans proved to be a significant technical barrier for further research and development. These roadblocks brought human stem cell research to a grinding halt, until the advent of induced pluripotent stem cells (iPSCs).

## Induced pluripotent stem cells

The pioneering work by Takahashi and Yamanaka (2006), Takahashi et al. (2007) and Yu et al. (2007), which showed that forced expression of four-transcription factors could convert somatic cells into pluripotent stem cells (Takahashi and Yamanaka, 2006; Takahashi et al., 2007; Yu et al., 2007) has been lauded as a major biological discovery of the twenty first century. The ability of these iPSCs to differentiate into any somatic cell type, just like ESCs but without their ethical concerns, have triggered an explosion of interest in their clinical applications (Wu and Hochedlinger, 2011; Sayed et al., 2016). Indeed, patient-specific iPSCs can be created and differentiated to a particular cell type while still retaining the same genetic background. This has allowed researchers to delineate underlying mechanisms of disease by recreating “disease-in-a-dish” models that are specific to that particular individual (Park et al., 2008; Carvajal-Vergara et al., 2010; Marchetto et al., 2010; Moretti et al., 2010; Sun et al., 2012; Lan et al., 2013; Wu et al., 2015). Furthermore, these models can then be screened for new drugs in a high-throughput manner without exposing the patient to any risk (Matsa et al., 2011, 2014; Wang et al., 2014). For example, lab-grown patient-specific tissues can be subjected to different treatments, like a virtual clinical trial without the need to administer a single drug to the patient (Matsa et al., 2016; Sharma et al., 2017).

To date, iPSCs have 3 major applications in disease modeling, drug discovery, and regenerative medicine, and thus have attracted enormous scientific interest (Sayed et al., 2016; Sayed and Wu, 2017). Much effort has also been exerted to determine the molecular mechanisms of iPSC generation in order to improve efficiency and make them safer. This has led to the identification of many barriers and enhancers in the reprogramming process. For examples, several transcription factors (Gli-similar 1, Forkhead box protein H1 and Bright/AT rich interactive domain 3A), signaling pathways (Transforming growth factor beta, Wnt/β-catenin, Hippo and p53), and epigenetic modifiers (Methyl-CpG-binding domain protein 3, Disruptor of telomeric silencing 1-like, Histone deacetylase, DNA methyltransferase, and histone demethylases) have been identified that behave either as facilitators or as barriers to the reprogramming process (Ebrahimi, 2015).

## Transdifferentiation

The iPSC technology has practical applications in the development of disease-specific cells for understanding pathobiology and for drug screening (Yamanaka and Blau, 2010). However, there are limitations to the application of iPSC-derived cells for therapy, including the risk of teratoma and the delayed process of differentiating them to therapeutic cells. Based on the initial discovery by Yamanaka and colleagues, another concept that has re-emerged is the direct differentiation of one somatic cell to another somatic cell without passing through the pluripotent state, a process known as “transdifferentiation.” For patient-specific cell therapy, transdifferentiation can be an attractive alternative approach, as the derived cells can be differentiated at a much faster rate, avoiding the risk of teratoma formation. Indeed, several groups, including ours, have been successful in directly converting fibroblasts to neurons (Vierbuchen et al., 2010), cardiomyocytes (Ieda et al., 2010), or endothelial cells (Ginsberg et al., 2012; Margariti et al., 2012). However, safety remains a concern, as most of the transdifferentiation protocols require the introduction of viral vectors encoding transcription factors. Although effective in inducing transdifferentiation, these viral vector-mediated transcription factors cannot be used for clinical applications. To circumvent these safety issues, many researchers have adopted a small molecule approach toward transdifferentiation in which chemical cocktails are used instead of viral vector-derived factors (Cheng et al., 2014; Fu et al., 2015; Sayed et al., 2015). Even though, this has been a giant step toward the clinical application of stem cells, a major drawback of transdifferentiation is the difficulty to obtain sufficient number of target cells that can replace the diseased tissue. This has led many researchers to explore the body's own regenerative machinery to replace the damaged tissues. One such system that has lately received much attention is the immune system and its contribution toward regeneration of damaged tissues.

## Patient, heal thyself

Humans possess an innate ability to heal following injury or tissue damage, either by activating dormant resident precursor cells or by forming a scar. This process of tissue regeneration requires a coordinated effort by the immune system to remove the cellular debris, activate the progenitor cells, and facilitate angiogenesis in the regenerated tissue. Immune cells, such as inflammatory monocytes and macrophages have been implicated as key regulators in tissue repair and regeneration (Arnold et al., 2007; Epelman et al., 2014). For example, lower vertebrates, such as amphibians and fish have been shown to regenerate their entire limbs, heart and even their brain by modulating the immune system via defined recruitment of macrophages (Kyritsis et al., 2012; Godwin et al., 2013; Petrie et al., 2014). Similarly, neonatal mice also possess this unique ability to regenerate the heart following injury by recruiting macrophages which provide the necessary cues for cardiomyocyte proliferation and angiogenesis (Porrello et al., 2011; Aurora et al., 2014). However, as adults, mammals have lost this regenerative capacity due to the maturation of their immune system. Indeed, many studies have shown that in mammals, an inverse relationship exists between the capacity to regenerate and the maturity of the immune system (Fukazawa et al., 2009; Mescher et al., 2013), thereby suggesting that the immune system impairs mammalian regenerative capacity. In this review paper, we aim to highlight the intricacies of the human innate immune system and its role in nuclear reprogramming and regeneration.

## “Toll” on the road to nuclear reprogramming

The original mouse and human iPSCs were generated by either retroviral or lentiviral transduction of transcription factors (Takahashi and Yamanaka, 2006; Takahashi et al., 2007). Although these transgenes are silenced after reprogramming, they can be unintentionally reactivated, thereby increasing the risk of tumorigenicity. To avoid these unintentional drawbacks, there has been a concerted effort by the research community to generate safer iPSCs by using non-genetic methods, such as use of excisable vectors (Carey et al., 2009; Woltjen et al., 2009), non-integrating vectors (Wernig et al., 2008; Jia et al., 2010), plasmid vectors (Okita et al., 2008; Yu et al., 2009), recombinant proteins/peptides (Zhou et al., 2009; Lee et al., 2012), and most recently, only small molecules (Hou et al., 2013). Though safer, these methods of reprogramming have very low yields. Moreover, despite their tumorigenic properties, viral-based reprogramming is still considered to be the most reproducible and reliable method for iPSC generation. To improve the overall low efficiency of generating iPSCs with most non-integrating approaches, it is crucial to understand the molecular mechanisms of viral-induced reprogramming.

**Figure 1:**
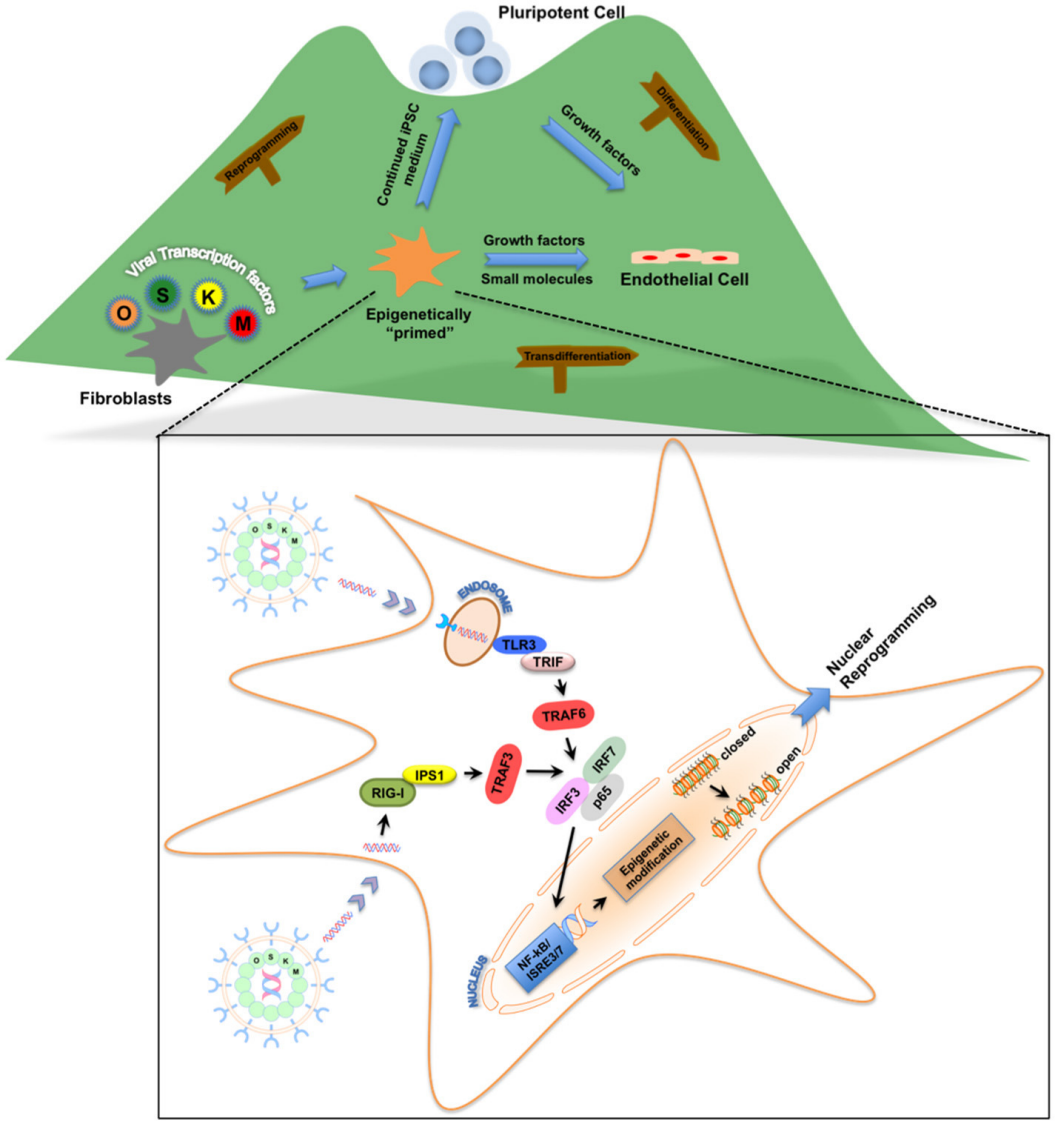
Roadmap for the role of innate immunity in nuclear reprogramming. Nuclear reprogramming works via manipulation of networks that govern an epigenetic state. Viral transcription factors can reprogram fibroblasts by moving the cells to the top of the “mountain” in the “epigenetic landscape.” Once these fibroblasts are “epigenetically primed” and reach an intermediate plastic state, they can be pushed toward pluripotency or transdifferentiated to endothelial cells. Inset: Activation of innate immunity via Toll-like receptors or RIG-I-like receptors by these viral transcription factors enables the fibroblasts to becoming “epigenetically primed” by changing the status of the chromatin structure from a “closed” conformation to an “open” conformation.

In our quest to reprogram human fibroblasts to iPSCs using cell-permeant peptides (CPPs) as an attempt to yield safer iPSCs, we serendipitously discovered that innate immune signaling plays a critical role in viral-based reprogramming via the activation of toll-like receptor 3 pathway (TLR3) (Lee et al., 2012). Multiple failed attempts to reprogram human fibroblasts using CPPs led us to compare downstream gene expression pattern between viral- and CPP-based delivery methods of reprogramming. The differential patterns of gene expression induced by viral constructs compared to CPPs led us to hypothesize that the viral particles itself may contribute to reprogramming. Indeed, loss- and gain-of-function studies confirmed that knockdown of TLR3 in human fibroblasts impaired nuclear reprogramming, while addition of polyinosinic:polycytidylic acid (poly I:C), a TLR3 agonist enhanced CPP-induced nuclear reprogramming. Importantly, we discovered that viral particles or poly I:C via activation of TLR3 can regulate the chromatin structure by changing the status from a “closed” conformation to an “open” conformation, thereby allowing the reprogramming factors to regulate associated genes (Figure 1). Lastly, we found that the effects of innate immunity on nuclear reprogramming were also mediated by the nuclear factor-κB (NF-κB) and interferon regulatory factor 3 (IRF3). Since our discovery, the notion that inflammatory pathways play a role in reprogramming has gained more traction. Recently, two groups found that a pro-inflammatory molecule, interleukin-6 (IL6), is key for *in vivo* reprogramming (Chiche et al., 2016; Mosteiro et al., 2016). Both these studies found that expression of reprogramming factors in living tissues results in cellular damage and the release of IL6, which then positively regulates efficient reprogramming of neighboring cells.

Our initial work focused on delineating the involvement of TLR3 in nuclear reprogramming; however pattern recognition receptors (PRRs), in addition to membrane-bound TLRs, also include cytoplasmic receptors (Roy and Mocarski, 2007). These cytoplasmic PRRs are also known to recognize viral double- and single-stranded RNA via a group of RNA helicases which include retinoic acid-inducible gene I (RIG-I) and melanoma differentiation-associated protein 5 (MDA5) collectively referred to as RIG-I-like receptors (RLR) (Yoneyama et al., 2004; Kato et al., 2005). As these PRRs recognize viral dsRNA and signal via NF-κB and IRF3, they could also be expected to enhance nuclear reprogramming. Based on this, we hypothesized that these cytoplasmic proteins, which regulate inflammatory and apoptotic responses could also enhance nuclear reprogramming. Our hypothesis was further strengthened when our attempt to reprogram fibroblasts from TLR3-knockout mice failed to completely abolish nuclear reprogramming, thereby suggesting that other pathways might also be involved (Sayed et al., 2017). Knockdown of the RLR pathway partially inhibited reprogramming, while addition of the RLR ligand enhanced the process, suggesting that RLR pathways are yet another PRR that might be involved in viral-based nuclear reprogramming (Sayed et al., 2017). It could be expected that other TLRs or PRRs that respond to inflammatory signals via NF-κB could also play an important role in nuclear reprogramming.

## “Toll” on the road to transdifferentiation

During this flurry of activity in the iPSC field following its discovery, another concept that re-emerged was the direct reprogramming of somatic cells to another desired cell type. Indeed, transdifferentiation of somatic cells was first described many years before the discovery of iPSCs when MyoD, a mammalian transcription factor, was found capable of converting fibroblasts to myocytes (Davis et al., 1987). Since then, many researchers have overexpressed lineage-specific transcription factors to convert somatic cells to their choice of cell. Transdifferentiation of fibroblasts to a variety of cells, such as cardiomyocytes (CMs) (Ieda et al., 2010), endothelial cells (ECs) (Ginsberg et al., 2012; Margariti et al., 2012), neurons (Vierbuchen et al., 2010), and hepatocytes (Huang et al., 2011) has now been successfully achieved. Similar to reprogramming, transdifferentiation requires the introduction of viral vectors that encode transcription factors, which then manipulate the inaccessible chromatin for lineage factors to bind and execute their function (Kelaini et al., 2014). Alternatively, transdifferentiation could also be achieved via induction of an intermediate plastic state by briefly expressing pluripotency transcription factors, such as Oct4 followed by lineage-specific differentiation cues (Wang et al., 1999). Termed “cell-activation and signaling-directed” (CASD) lineage conversion, this transdifferentiation method still utilized integrating approaches to overexpress transgenes. However, it may be more safe and efficient to have an integration-free approach for induction of the CASD lineage conversion, using small molecules that can suspend the cells in a plastic state, thereby making them responsive to the microenvironment cues for transdifferentiation.

With the recognition of the role of innate immunity in nuclear reprogramming and its directed manipulation to favor an open chromatin state, we hypothesized that this cell state might increase phenotypic plasticity and facilitate transdifferentiation. The value of innate immune signal transduction in establishing epigenetic plasticity of iPSCs led to our discovery that activation of TLR3, together with adequate microenvironmental cues that drive EC-specification, may be sufficient to induce the transdifferentiation of human fibroblasts into induced endothelial cells (iECs) (Figure 1; Sayed et al., 2015). This strategy included a TLR3 agonist combined with small molecule compounds that trigger the EC differentiation pathway to yield bona fide ECs. Human iECs have been previously generated by the forced expression of EC-specific transcription factors. However, our findings suggested that manipulation of innate immune signaling was able to induce epigenetic plasticity in fibroblasts to transdifferentiate them into functional iECs. We believe that this form of chemical transdifferentiation does not include any genetic modification when compared to the other two forms, and thus has greater potential for clinical application (Sayed et al., 2013).

## Conclusion and future perspectives

Since the initial discovery by Dr. Shinya Yamanaka that overexpression of a few transcription factors could reprogram mouse and human fibroblasts to stem cells, the iPSC technology has expanded rapidly toward the three major applications: disease modeling, drug discovery, and regenerative medicine. The last decade has seen a significant amount of effort invested to determine the molecular mechanisms of iPSC generation with the goal to make iPSC technology safe and efficient. In an effort to make iPSC generation safe, we discovered that innate immunity via the activation of TLR3 or RIG1 is required for efficient nuclear reprogramming (Lee et al., 2012; Sayed et al., 2017). Moreover, our subsequent study showed that by precisely manipulating the innate immune system in an appropriate microenvironmental milieu, we could help transdifferentiate one somatic cell type to another desired somatic cell type (in our case, human fibroblasts to endothelial cells), without an intermediate iPSC stage (Sayed et al., 2015).

With the growing appreciation of the role of the vertebrate immune system in the regeneration of damaged tissues, we believe that by utilizing the good and by inhibiting the bad of the inflammatory pathways, immune-mediated regeneration with the use of small molecules could push the existing stem cell therapies toward clinical application. In the future, understanding the mechanisms involved in innate immunity-mediated reprogramming can help us better understand the complexities of tissue repair and regeneration.

## Author contributions

CL: wrote the initial draft of the manuscript, FH: wrote the initial draft of the manuscript, NS: conception and design of the manuscript, financial support, administrative support; manuscript writing; final approval of manuscript.

## Disclosures

NS is an inventor of the intellectual property, assigned to Stanford University related to the use of innate immune signaling for nuclear reprogramming and transdifferentiation. The other authors have no disclosures.

### Conflict of interest statement

The authors declare that the research was conducted in the absence of any commercial or financial relationships that could be construed as a potential conflict of interest.
